# Assessing the Impact of Fencing on Postural Parameters: Observational Study Findings on Elite Athletes

**DOI:** 10.3390/sports12050130

**Published:** 2024-05-13

**Authors:** Giulia Di Martino, Marco Centorbi, Andrea Buonsenso, Giovanni Fiorilli, Carlo della Valle, Enzo Iuliano, Giuseppe Calcagno, Alessandra di Cagno

**Affiliations:** 1 Department of Medicine and Health Sciences, University of Molise, 86100 Campobasso, Italy; giulia.dimartino21@gmail.com (G.D.M.); marco.centorbi@hotmail.it (M.C.); andrea.buonsenso@unimol.it (A.B.); fiorilli@unimol.it (G.F.); giuseppe.calcagno@unimol.it (G.C.); 2 Department of Neurosciences, Biomedicine and Movement, University of Verona, 37134 Verona, Italy; 3 Faculty of Psychology, eCampus University, 22060 Novedrate, Italy; 4 Department of Movement, Human and Health Sciences, University of Rome “Foro Italico”, 00135 Rome, Italy; alessandra.dicagno@uniroma4.it

**Keywords:** postural asymmetries, postural control, stabilometry, postural tone, elite fencers

## Abstract

The aim of the study was to evaluate whether the static stabilometric parameters among elite fencers, were affected by prolonged, asymmetric training regimen. A sample of 26 elite fencers of both genders, aged 19.15 ± 2.24 years, practising one of the three disciplines foil, épée, or sabre, was recruited for the study. Anthropometric measurements including thigh and calf circumferences and postural assessment based on the weight distribution on a stabilimeter platform were performed. Postural tone, as indicated by measures such as sway length and sway area ratio was calculated.. No notable anthropometric asymmetries were detected within the examined group The weight distribution patterns on the support quadrants in static stabilometric measurements did not suggest clinically significant issues. There were no significant differences among subgroups based on gender and lower limb dominance for both anthropometric and stabilimeter variables. However, 30.8% of participants showed anomalies in postural tone (hypertonic and hypotonic condition). Five out of eight athletes found with abnormal postural tone were foil fencers, suggesting a potential discipline-specific effect. Individual adjustments were found in foil fencers. These findings provide insights into the potential effects of fencing training on postural parameters among elite athletes.

## 1. Introduction

Physical exercise and sports practice constitute a continuously evolving field of research, focused on understanding the specific effects of different sports activities on physiological and biomechanical parameters. Baropodometry, which focuses on measuring and analysing the centre of pressure (COP) of the feet, is widely acknowledged as a crucial component for evaluating postural balance across different populations [1,2,3,4].

In the context of this research, the evidence indicates that controlling postural asymmetries represents a preventive factor for the health and life of individuals, alongside their sports performance. For instance, strength asymmetries appear to have a detrimental effect on performance tasks such as lateral movements, jumps, and specific sports skills such as kicking accuracy [5]. Furthermore, a significant relationship has been identified between static and/or dynamic balance and performance metrics in different sports disciplines like archery, golf, baseball, ice hockey, tennis, and snowboarding [6].

In elite fencing, successive performance depends on precise asymmetrical movements [7], whereas the success of athletes depends greatly on executing technically proficient and powerful one-sided lunges [8,9]. During these lunges, upper limb movement is followed by the extension of the front knee, corresponding to the dominant (D) leg, and the vigorous lengthening of the back leg, e.g., the non-dominant (ND) leg, during the propulsion phase [10]. A previous study determined that the extensor muscles of the ND leg play a crucial role in generating power during the propulsive (concentric)phase. Conversely, extensor muscles of the D leg are responsible for the “braking” (eccentric phase) and stabilisation of the leg during lunge execution. These significant differences between actions and movements induce differential mechanical stresses between the front and rear legs during attacking lunges, resulting in strong bilateral asymmetries between the two legs [11]. However, there is limited and inconclusive data regarding potential disparities in strength and balance between the lower limbs of fencers, particularly among young athletes [12].

The present study aims to contribute to this area of research, with the main objective being to assess whether regular fencing practice influences static stabilometric parameters of elite practitioners with at least 12 years of experience, focusing on identifying long-term adaptations in postural control and understanding how repetitive specific kinetic patterns of this sport may alter the distribution of body weight across different support quadrants.

The goal could be to address current gaps in scientific understanding and provide practical information useful for athletes and coaches in designing targeted training programs.

## 2. Materials and Methods

### 2.1. Study Design

The present study is a cross-selectional observational design to assess whether a long training adaptation in an asymmetric sport, such as fencing, could adversely impact the quality of balance and postural stability in elite athletes, as well as to detect potential alterations in a bi-podalic station.

### 2.2. Participants

This study involved a population of 26 elite fencers of both genders who have participated in recent years in World Cups and European Championships. The participants of both genders (18 males and 8 females) are aged 19.15 ± 2.24 years. The initial sample divided into subgroups bases on gender, specialization (weapon), and lower limb dominance for further examination.

Concerning the disciplines, 10 participants were sabre fencers, 9 were foil fencers, and 7 were epee fencers. Furthermore, 19 participants were right-footed, while 7 participants were left-footed. The inclusion criteria used for the enrolment were (1) age between 18 and 23 years; (2) a history of high-level training experience of at least 12 years; (3) engaging in one of the three fencing disciplines (foil, épée, or sabre); and (4) a training frequency of at least sessions per week. The exclusion criteria were (1) injuries that occurred in the previous 3 months; (2) participation or involvement in training other than that one related to their fencing activity; and (3) use of drugs, medications, or other conditions that could influence the test results or that could have introduced a bias into the study. The characteristics of the sample are reported in Table 1.

All participants were informed about the objective and procedures of the study and signed the informed consent. The study was designed and conducted by the Declaration of Helsinki and fully approved by the Bioethical Local Committee of the University of Rome “Foro Italico” (University Committee for Research—CAR-IRB—Code: CAR 15/2019).

### 2.3. Procedures

#### 2.3.1. Assessment of Anthropometric Data

The anthropometric parameters included weight, height, thigh, and calf circumferences of both legs of each participant. To assess asymmetries between the dominant and non-dominant lower limbs, the percentual differences between dominant thigh and calf circumferences and non-dominant circumferences were also assessed. Body height was measured using a metal anthropometer to the nearest 0.1 cm (mod. Harpenden Portable Stadiometer 603 VR; Holtain Ltd., Crosswell, UK). Body weight was measured with minimal clothing to the nearest 0.1 kg with a medical electronic balance (mod. A & D UC-352BLE wireless balance; A & D Instruments Ltd., Abingdon, UK); body mass index (BMI) was also calculated as body weight (kg) divided by squared body height (m2). For thigh and calf circumferences, a flexible calibrated tape was used, and measurements were recorded to the nearest 0.1 cm (mod. Seca 201; Seca Ltd., Hamburg, Germany). Three measurements were recorded, and the mean values were used for the analysis.

#### 2.3.2. Postural Assessment

Postural stability was evaluated by recording the plantar pressure distribution and quantifying the displacement of the COP using a stabilometric platform (FDM-S, Zebris Medical GmbH, Isny, German). The postural parameters obtained using the Zebris platform were considered for statistical analysis (Table 2).

The assessment of the postural parameters on the stabilometric platform was repeated three times, with a two-minute break between repetitions for each athlete. The mean of these three evaluations was calculated and considered for the statistical analysis. The assessment on the platform lasted 52 s and, and during this time, the participants had to maintain a standing position on both feet with open eyes and with arms by their sides. To prevent extra movements, the participants were asked to keep their gaze fixed in front of them, fixating on a black dot, 2 cm in diameter, placed at eye level, on a white wall at a 2 m distance. The assessment of the postural parameters was conducted in a quiet room without lights, sounds, or other factors that could distract the participants or influence the measurements.

### 2.4. Statistical Analysis

The normal distribution of continuous variables was verified using the Shapiro–Wilk test. A descriptive statistical analysis (mean ± SD) of the collected data was conducted for all the participants considered as a single group and successively divided into different subgroups based on gender, dominance, and practised discipline.

Subsequently, the percentual differences of the thigh and calf circumferences of the dominant vs. non-dominant leg of each participant were evaluated to assess whether this percentual difference could be indicative of clinically significant asymmetry when greater than 10% (Table 1) [13]. The percentual distribution of the body weight on the 4 quadrants of the stabilometric platform was investigated to evaluate the possible presence of clinically significant postural alterations (i.e., repartition of the body weight between the left and right quadrants >10%) [14]. Concerning the postural parameters, the ratio sway length/sway area (SL/SA) for each participant was also assessed and compared with normative reference data to evaluate the possible presence of clinically significant postural alterations: this ratio is proportional to postural tone and a value under 1.5 can suggest a hypertonic postural tone, whilst a value over 3 can suggest a hypotonic postural tone [15].

Finally, to verify significant differences concerning anthropometric and postural parameters between genders, right-footed/left-footed, and/or practised disciplines, an analysis of variance (ANOVA) was performed comparing the different subgroups: male vs. female, right-footed vs. left-footed, and foil vs. épée vs. sabre. The dependent variables analysed were body weight, body height, percentual differences between dominant and non-dominant thigh and calf circumferences (anthropometric variables), SL, SA, SL/SA, and SV (postural variables). In the comparisons among foil vs. épée vs. sabre, due to the presence of 3 subgroups, in the case of statistically significant values in ANOVA, a pairwise comparison among the 3 disciplines was conducted using Bonferroni post-hoc.

A *p*-value < 0.05 was considered statistically significant for all the analyses. Analyses were conducted using SPSS software version 28 (IBM Inc., Chicago, IL, USA).

## 3. Results

The results of the descriptive analyses are reported in Table 3. Data showed that the percentual differences of thigh and calf circumferences of the dominant vs. non-dominant leg did not suggest anthropometric asymmetry since the percentual differences respectively ranged between −8.47% and 2% for thigh circumferences (mean ± SD = −3.47% ± 2.13), and between −5.13% and 3.33% for calf circumferences (mean ± SD = 0.17% ± 1.59).

The data relating to weight distribution on feet did not highlight any anomalous situations that could have suggested a problem of clinical interest. The values are substantially in line with those of scientific literature referring to a general population [14]. These data were graphically reported in Table 3.

**Figure 1:**
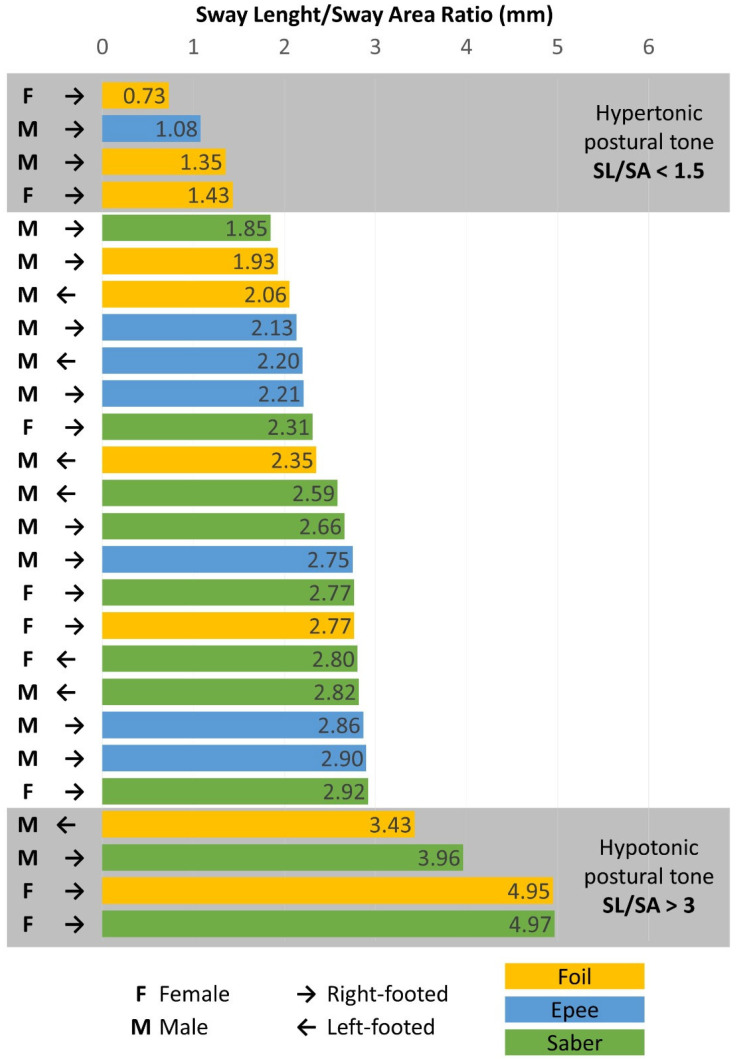
Distribution of sway length and sway area ratio for each evaluated athlete, classified by gender, handedness, and fencing weapon type.

Concerning the SL/SA ratio, the data showed that four participants had a value of <1.5, indicating a hypotonic postural tone, while the other four participants had a value of >3, indicating a hypertonic postural tone. In total, 8 of the 26 participants (30.8% of the sample) showed an anomaly in the postural tone. In particular, five of the eight participants with an SL/SA value of <1.5 or >3 were foil fencers, and only one of the eight was left-footed. The values of the SL/SA of the 26 participants were graphically reported in Figure 1.

The ANOVA did not show significant differences in the anthropometric variables or the stabilometric variables between the considered subgroups (male vs. female, right-footed vs. left-footed, and foil vs. épée vs. sabre). The results are reported in Table 3.

## 4. Discussion

This study aimed to assess whether the balance and postural control of elite athletes with at least 12 years of experience were influenced by regular and long-term fencing practice, with no significant alterations observed in postural control and balance parameters. The present study results showed no substantial alteration in balance and postural stability parameters in the examined fencers, despite the asymmetrical requirements of the sport, which may lead to asymmetric adaptations [16]. Despite fencing being classified as an asymmetric sport, in our sample, the difference in the thigh and calf circumferences of the lower limbs, from a clinical standpoint, fell within a normal range, considering that the disparity between the dominant and non-dominant lower limb did not exceed 10–15% [17].

Although no asymmetry was observed in the anthropometric measurements, we deemed it essential to assess the load distribution on the support quadrants in static stabilometric measurements. The results confirmed that the low difference in distribution between the dominant and non-dominant lower limb fell within the normal range, consistent with findings from previous studies [11]. This result could be due to the evolution of methodologies adopted by some high-level athletes, which currently involves the integration of compensatory exercises into predominantly unilateral specific training [18,19]. 

Referring to the length and area of COP displacement, the examined sample showed a slightly higher-than-normal increase in COP length. Likely, the fencing technique has induced specific adaptations. In fencing the guard position and the structure of lunge required movements that involve restricted visual control for balance, thereby stimulating an increase in postural tone and reflexes, particularly within the lower limb mechanoreceptors, which are assessed at a postural level in terms of the centre of pressure (COP) length and speed [7]. Previous studies have shown that an increase in the length of COP displacements is an indicator of enhanced information flow, and an increase in the area means a deterioration in postural control, leading to statistically significant disturbances in balance ability [14]. Moreover, fencers use visual feedback minimally due to the head position resulting in a 90° rotation, with their attention fully focused on monitoring the opponent’s actions; moreover, fencing techniques stimulate the otoliths and the vestibular system [20].

Regarding the COP area, the extreme variability of the context in which the “fencing phrase” takes place destabilised the athlete’s balance, inducing continuous postural adjustments that broaden the COP area [21]. It is evident how fencers constantly train their spatial orientation ability in a variable environment compared to athletes from other sports [22], motivating an increase in the COP area.

No statistically significant differences in athletes, both in terms of gender and lateral dominance, were found despite prior studies highlighting differences in technical-tactical approaches between men and women in fencing [23].

Nevertheless, 8 out of 26 elite athletes (about 30% of the sample) showed postural tone values identifiable by the sway length and sway area ratio (SL/SA), different from the reference values suggested by Gallamini and colleagues (2021). In particular, four athletes exhibit hypertonic postural behaviour, while the other four exhibit hypotonic behaviours. The value of SL/SA was out of the normative reference range, suggesting an anomalous relationship between the SL and SA of the COP displacement [15]. A hypertonic postural behaviour with an SL/SA ratio exceeding 1.5 leads to increased energy expenditure, which could negatively influence performance and fatigue, conversely, with participants showing a hypotonic behaviour. Moreover, those with a ratio greater than 3 showed increased COP movements (area) despite low energy expenditure.

However, the out-of-range postural behaviours of the eight athletes could be due to individual adaptations only partially related to their sports activity. It would be considered that balance strategies are often individualised [24], and each athlete shows different postural control characteristics [25]. 

We would have expected to find that other stabilometric and anthropometric parameters, such as SL, SA, SV, weight distribution across quadrants, and calf and leg circumferences, would be anomalous values. However, these additional parameters did not yield significant anomalous values. This suggests that hypertonic or hypotonic behaviours may be non-pathological and individualised adaptations.

The differences in hypotonia and hypertonia postural conditions among foil fencers, sabre fencers, and épée fencers can be attributed to various factors, including the demands of the discipline’s biomechanical characteristics of movement and specific training. The different techniques and strategies employed in specific actions within the fencing disciplines can influence the muscular and postural development of practitioners. Foil fencing, due to its smaller target area, necessitates more precise and controlled movements compared to the other two specialities. Thus, foil training could favour different postural conditions, characterised by reduced or increased muscle activity. The rapid and coordinated movements required in foil fencing to land a valid thrust may promote hypertonia postural conditions. Conversely, in épée and sabre fencing, which involve broader and more dynamic movements, due to the larger target area, fewer postural control adjustments are required than in foil fencing. Despite the fact that épée fencing does not typically involve dynamic movements as those in sabre fencing, the styling emphasis on strategic positioning and precision contributes to similar muscular adaptations and potential postural differences.

Furthermore, the fencing tactics of foil, which involve multiple changes in direction for both defence and attack, can influence posture and weight distribution. Foil fencers tend to assume a more upright position to maintain greater accuracy in thrusts, unlike sabre fencers and épée fencers, who adopt a more frontal torso position to generate greater power in their attacking actions [26].

## 5. Limitation

A small sample size does not allow us to have clear and unequivocal evidence and our statements could be considered speculative.

This study had a numerically limited sample, which could represent a limitation to the reliability of the results.

## 6. Conclusions

The study aimed to investigate whether long-term training in an asymmetric sport had induced alterations in the postural control and balance of elite athletes. The results highlighted that some alterations, both in the muscular asymmetry in the lower limbs of fencers and in the stabilometric parameters, fell within a range of normality from a clinical standpoint and did not have negative consequences for static balance and postural control in elite fencing athletes. Probably, these results were achieved through conditioning management and a meticulously tailored increase in compensatory load for high-level athletes. Each training intervention may be tailored taking into account individual postural control strategies, given that these strategies vary from athlete to athlete and from specialty to specialty (foil, epee, sabre).

Further studies are needed focusing on the lower limbs’ different kinetic characteristics, which may lead to asymmetric postural adaptations and individualised training interventions.

## Figures and Tables

**Table 1:** Characteristics of the sample.

	Age (Years)	Weight (Kg)	Height (m)	BMI (Kg/m^2^)	Thigh Right (cm)	Thigh Left (cm)	Difference of Thigh (%)	Calf Right (cm)	Calf Left (cm)	Difference of Calf (%)
Sample (*n* = 26)	19.15 ± 2.24	67.65 ± 11.13	1.77 ± 0.09	21.50 ± 2.57	54.93 ± 5.14	54.07 ± 4.45	−3.41 ± 2.40	35.25 ± 2.25	35.10 ± 2.15	−0.16 ± 1.75
Foil (*n* = 9)	19.56 ± 2.40	63.00 ± 9.25	1.74 ± 0.09	19.85 ± 2.06	54.69 ± 5.56	54.03 ± 4.86	−2.93 ± 2.10	35.19 ± 2.49	35.02 ± 2.57	−0.33 ± 1.81
Epee (*n* = 7)	17.14 ± 0.38 *	67.86 ± 5.87	1.82 ± 0.07	20.54 ± 2.50	56.64 ± 6.18	56.00 ± 5.55	−3.46 ± 3.68	35.36 ± 2.06	35.50 ± 2.03	0.26 ± 1.46
Sabre (*n* = 10)	20.20 ± 2.04	71.70 ± 14.71	1.78 ± 0.10	22.43 ± 3.27	53.96 ± 4.12	52.75 ± 2.96	−3.81 ± 1.62	35.22 ± 2.39	34.89 ± 2.02	−0.29 ± 2.00

* The epee fencers are significantly younger than foil and sabre fencers.

**Table 2:** Legend of postural parameters obtained using the Zebris platform and analysed in the present study.

Postural Parameter	Acronym	Description
Sway Length (mm)	SL	The length of the COP path during the test
Sway Area (mm^2^)	SA	The area of the surface swept by the radius connecting the mean COP to all subsequent path points
Sway Length/Sway Area Ratio (mm)	SL/SA	The ratio between Sway Length and Sway Area. This ratio is proportional to postural tone (a value under 1.5 suggests a hypotonic postural tone, whilst a value over 3 suggests a hypertonic postural tone)
Speed Variation (mm/s^2^)	SV	Average change in the velocity of the centre of pressure
Weight percentage on non-dominant-side quadrants (%)	% ND-S	The percentage of weight distributed on the non-dominant-side quadrants of the platform.
Weight percentage on dominant-side quadrants (%)	% D-S	The percentage of weight distributed on the dominant side of the platform.
Weight percentage on non-dominant-anterior quadrant (%)	% ND-ANT	The percentage of weight distributed over the non-dominant anterior of the platform.
Weight percentage on non-dominant-posterior quadrant (%)	% ND-POST	The percentage of weight distributed over the non-dominant-posterior quadrant of the platform.
Weight percentage on dominant-anterior quadrant (%)	% D-ANT	The proportion of weight distributed over the dominant-anterior quadrant of the platform
Weight percentage on dominant-posterior quadrant (%)	% D-POST	The proportion of weight distributed over the dominant-posterior quadrant of the platform.

**Table 3:** Stabilometric parameters of the sample as mean and standard deviation.

	Sample	Male	Females	Left-Handed	Right-Handed	Sabre	Epee	Foil
Parameter	Means ± SD	Means ± SD	Means ± SD	Means ± SD	Means ± SD	Means ± SD	Means ± SD	Means ± SD
Sway Area (mm^2^/s)	5.49 ± 4.14	5.09 ± 2.96	6.41 ± 6.22	4.39 ± 1.36	5.90 ± 4.75	3.72 ± 1.40	5.34 ± 3.06	7.59 ± 5.95
Sway Length (mm/s)	11.21 ± 2.45	11.09 ± 2.62	11.48 ± 2.17	11.01 ± 2.51	11.29 ± 2.49	10.25 ± 2.33	10.90 ± 1.94	12.53 ± 2.58
Sway Length/Sway Area Ratio (mm)	2.57 ± 1.00	2.56 ± 0.91	2.59 ± 1.24	2.61 ± 0.46	2.56 ± 1.15	2.97 ± 0.89	2.30 ± 0.63	2.33 ± 1.27
Variation in Speed (mm/s^2^)	68.10 ± 34.75	63.54 ± 33.77	78.72 ± 36.88	67.95 ± 32.42	68.31± 36.42	58.56 ± 30.6	59.22 ± 20.56	85.93 ± 43.15
Weight percentage on non-dominant-side quadrants (%)	48.74 ± 3.42	48.46 ± 3.53	49.38 ± 3.30	46.66 ± 2.54	49.51 ± 3.44	48.92 ± 2.63	49.67 ± 2.99	47.82 ± 4.51
Weight percentage on dominant-side quadrants (%)	51.05 ± 3.41	51.34 ± 3.52	50.4 ± 3.29	53.13 ± 2.57	50.28 ± 3.42	50.85 ± 2.60	50.16 ± 2.95	51.97 ± 4.54
Weight percentage on non-dominant-anterior quadrant (%)	23.39 ± 5.39	22.48 ± 4.72	25.44 ± 6.04	21.84 ± 3.61	23.96 ± 5.89	23.85 ± 4.90	21.36 ± 4.89	24.47 ± 6.37
Weight percentage on non-dominant-posterior quadrant (%)	25.35 ± 5.00	25.98 ± 4.72	23.94 ± 5.65	24.81 ± 2.59	25.55 ± 5.69	25.07 ± 4.22	28.31 ± 6.36	23.36 ± 3.93
Weight percentage on dominant-anterior quadrant (%)	25.81 ± 4.08	26.02 ± 3.77	25.35 ± 4.97	24.46 ± 3.38	26.31 ± 4.28	25.38 ± 3.19	26.31 ± 4.38	25.9 ± 5.07
Weight percentage on dominant-posterior quadrant (%)	25.24 ± 5.23	25.32 ± 5.33	25.05 ± 5.33	28.67 ± 4.62	23.97 ± 4.96	25.47 ± 4.16	23.84 ± 4.86	26.07 ± 6.76

## Data Availability

Data are available upon request to the corresponding author: due to ethical restriction.
